# Early treatment versus expectative management of patent ductus arteriosus in preterm infants: a multicentre, randomised, non-inferiority trial in Europe (BeNeDuctus trial)

**DOI:** 10.1186/s12887-018-1215-7

**Published:** 2018-08-04

**Authors:** Tim Hundscheid, Wes Onland, Bart van Overmeire, Peter Dijk, Anton H. L. C. van Kaam, Koen P. Dijkman, Elisabeth M. W. Kooi, Eduardo Villamor, André A. Kroon, Remco Visser, Daniel C. Vijlbrief, Susanne M. de Tollenaer, Filip Cools, David van Laere, Anne-Britt Johansson, Catheline Hocq, Alexandra Zecic, Eddy Adang, Rogier Donders, Willem de Vries, Arno F. J. van Heijst, Willem P. de Boode

**Affiliations:** 1 Department of Paediatrics, Division of Neonatology, Radboud university medical centre Nijmegen, Radboud Institute for Health Sciences, Amalia Children’s Hospital, Internal postal code 804, Geert Grooteplein Zuid 10, 6525 GA Nijmegen, The Netherlands; 2 Department of Neonatology, Academic Medical Centre Amsterdam, Emma Children’s hospital, Meibergdreef 9, 1105 AZ Amsterdam-Zuidoost, The Netherlands; 3 Department of Paediatrics, Division of Neonatology, Cliniques Universitaires de Bruxelles, Erasme Hospital, Route de Lennik 808, 1070 Brussels, Belgium; 4 Department of Paediatrics, Division of Neonatology, University Medical Centre Groningen, Beatrix Children’s Hospital, Hanzeplein 1, 9713 GZ Groningen, The Netherlands; 5 Department of Paediatrics, Division of Neonatology, VU University Medical Centre Amsterdam, De Boelelaan 1117, 1081 HV Amsterdam, The Netherlands; 6 Department of Neonatology, Maxima Medical Centre Veldhoven, de Run 4600, Postbus 7777, 5500 MB Veldhoven, The Netherlands; 7 Department of Paediatrics, Division of Neonatology, Maastricht University Medical Centre, P. Debyelaan 25, 6229 HX Maastricht, The Netherlands; 8 Department of Paediatrics, Division of Neonatology, Erasmus Medical Centre Rotterdam, Sophia Children’s Hospital, ‘s Gravendijkwal 230, 3015 CE Rotterdam, The Netherlands; 9 Department of Paediatrics, Division of Neonatology, Leiden University Medical Centre, Willem Alexander Children’s Hospital, Albinusdreef 2, 2333 ZA Leiden, The Netherlands; 10 Department of Paediatrics, Division of Neonatology, University Medical Centre Utrecht, Utrecht University, Wilhelmina Children’s Hospital, Lundlaan 6, 3584 EA Utrecht, The Netherlands; 11 Department of Paediatrics, Division of Neonatology, Isala Women’s and Children’s Hospital Zwolle, Dokter van Heesweg 2, 8025 AB Zwolle, The Netherlands; 12 Department of Neonatology, UZ Brussel – Vrije Universiteit Brussel, Laarbeeklaan 101, 1090 Brussels, Belgium; 13 Department of Paediatrics, Division of Neonatology, Antwerp University Hospital, Wilrijkstraat 10, 2650 Edegem, Belgium; 14 Department of Paediatrics, Division of Neonatology, Hôpital Universitaire des Enfants Reine Fabiola, Bruxelles, Jean Joseph Crocqlaan 15, 1020 Brussels, Belgium; 15 Department of Paediatrics, Division of Neonatology, Cliniques Universitaires St Luc, Avenue Hippocrate 10, 1200 Brussels, Belgium; 16 Department of Paediatrics, Division of Neonatology, Ghent University Hospital, De Pintelaan 185, 9000 Ghent, Belgium; 17 Department of Health Evidence, Radboud university medical centre, Geert Grooteplein Zuid 10, 6525 GA Nijmegen, The Netherlands

**Keywords:** Prematurity, Patent ductus arteriosus, Neonatal intensive care unit, Ibuprofen, Expectative management, Ductal ligation, Mortality, Necrotising enterocolitis, Bronchopulmonary dysplasia, Cost-effectiveness

## Abstract

**Background:**

Much controversy exists about the optimal management of a patent ductus arteriosus (PDA) in preterm infants, especially in those born at a gestational age (GA) less than 28 weeks. No causal relationship has been proven between a (haemodynamically significant) PDA and neonatal complications related to pulmonary hyperperfusion and/or systemic hypoperfusion. Although studies show conflicting results, a common understanding is that medical or surgical treatment of a PDA does not seem to reduce the risk of major neonatal morbidities and mortality. As the PDA might have closed spontaneously, treated children are potentially exposed to iatrogenic adverse effects. A conservative approach is gaining interest worldwide, although convincing evidence to support its use is lacking.

**Methods:**

This multicentre, randomised, non-inferiority trial is conducted in neonatal intensive care units. The study population consists of preterm infants (GA < 28 weeks) with an echocardiographic-confirmed PDA with a transductal diameter > 1.5 mm. Early treatment (between 24 and 72 h postnatal age) with the cyclooxygenase inhibitor (COXi) ibuprofen (IBU) is compared with an expectative management (no intervention intended to close a PDA). The primary outcome is the composite of mortality, and/or necrotising enterocolitis (NEC) Bell stage ≥ IIa, and/or bronchopulmonary dysplasia (BPD) defined as the need for supplemental oxygen, all at a postmenstrual age (PMA) of 36 weeks. Secondary outcome parameters are short term sequelae of cardiovascular failure, comorbidity and adverse events assessed during hospitalization and long-term neurodevelopmental outcome assessed at a corrected age of 2 years. Consequences regarding health economics are evaluated by cost effectiveness analysis and budget impact analysis.

**Discussion:**

As a conservative approach is gaining interest, we investigate whether in preterm infants, born at a GA less than 28 weeks, with a PDA an expectative management is non-inferior to early treatment with IBU regarding to the composite outcome of mortality and/or NEC and/or BPD at a PMA of 36 weeks.

**Trial registration:**

This trial is registered with the Dutch Trial Register NTR5479 (registered on 19 October 2015), the registry sponsored by the United States National Library of Medicine Clinicaltrials.gov NCT02884219 (registered May 2016) and the European Clinical Trials Database EudraCT 2017–001376-28.

## Background

Controversy exists about the optimal management of a patent ductus arteriosus (PDA) in preterm infants, especially in those born at a gestational age (GA) less than 28 weeks, due to a lack of evidence for any specific treatment including non-intervention [1–12]. There is also no consensus about the diagnostic criteria of a haemodynamically significant PDA (hsPDA). The reported incidence of a PDA in preterm infants is 30–60%, depending on the used definition, the timing of the diagnosis and the studied population.

PDA has been associated with mortality and major morbidities, such as bronchopulmonary dysplasia (BPD), pulmonary haemorrhage (PH), intraventricular haemorrhage (IVH), necrotising enterocolitis (NEC) and retinopathy of prematurity (ROP). The underlying pathophysiologic mechanism of this might be that a PDA with significant left-to-right shunting results in pulmonary hyperperfusion and systemic hypoperfusion, although any evidence for a causal relationship is lacking [13–19].

There is a large variation in the management of a PDA between centres [20–22]. Pharmacological closure of the PDA is most often attempted by inhibition of prostaglandin synthesis with non-selective cyclooxygenase inhibitors (COXi), such as indomethacin (INDO) or ibuprofen (IBU). By postponing the start of treatment of a PDA, the risk of redundant adverse effects of COXi is decreasing as the postnatal age (PNA) at which COXi is started increases, while the time of exposure to a hsPDA might be prolonged. Some reports suggest that a high dose of IBU might be more effective in ductal closure in preterm infants, especially in those less than 27 weeks’ gestation [23–26]. However, in a recent systematic review Ohlsson et al. refrained from recommendations regarding high dose IBU because of the limited number of patients enrolled in the studies [17]. Use of paracetamol has been associated with closure of a PDA in studies with only a limited number of preterm infants [27–35]. Moreover, the high dose of paracetamol (60 mg/kg/day) that is used to close the PDA gives rise to concerns about safety in preterm infants [36–38]. Standard ligation after failure of medical closure resulted in an increased incidence of BPD and neurodevelopmental impairment in comparison with delayed ligation in a selected population [39, 40]. Of interest, an expectative approach after failure of treatment was followed by ‘spontaneous’ closure in 67–86% of the patients [39, 41, 42].

Roughly, there are four different management approaches for preterm infants with a PDA: (1) prophylactic treatment; (2) pre-symptomatic (‘early’) treatment; (3) symptomatic (‘late’) treatment and; (4) expectative management [9, 12].

1. Prophylactic treatment consists of administration of COXi in all patients within a predefined patient group at a PNA less than 24 h. Prophylactic administration of INDO has been shown to reduce the incidence of symptomatic PDA, need for surgical ligation, and severe cerebral haemorrhage, and it seems to reduce the risk of PH [14, 43]. However, no effect was found on mortality or neurodevelopmental outcome at the age of 18–36 months [44]. Prophylactic IBU administration reduced the need for additional treatment of the PDA, but no effect has been described on the incidence of severe comorbidity [16].
2. Pre-symptomatic treatment is usually timed within the first 3 to 5 days of life. Significant left-to-right shunting can already occur early after birth, whereas clinical signs generally manifest later, with an average delay of 2 days [45, 46]. Echocardiography is used to identify patients with a potentially increased risk of PDA-associated morbidity [47]. No beneficial effects on relevant neonatal morbidity were found in a systematic review of the administration of INDO for asymptomatic PDA in preterm infants [13].
3. In symptomatic treatment, physicians wait for a possible spontaneous closure of the ductus arteriosus (DA). Treatment is only started when clinical signs and symptoms presumably related to a PDA develop. As formulated by Evans *‘It is the clinical approach that is most widely used but we do not have any evidence to support it’* [9].
4. Expectative management is characterized by ‘watchful waiting’ without the intention to actively close the DA. This approach is based on the fact that in a substantial portion of preterm infants the DA will close spontaneously [9, 41, 42, 48–50] and that there is a lack of proven benefit of medical treatment [1–12]. This expectative approach to a PDA in preterm infants is gaining interest. A recent multicentre retrospective study in 28,025 very low birth weight infants (< 1500 g) showed that the annual rate of patients who were not treated for their PDA (*n* = 12,002) increased from 60.5% in 2008 to 78.3% in 2014 [51].

### Meta-analysis of randomised controlled trials evaluating PDA treatment

We searched for all randomised controlled trials (RCTs) evaluating PDA treatment in the US National Library of Medicine (Medline), Cochrane Library, EMBASE and ClinicalTrials.gov database, using the Mesh terms: ‘infant, newborn’ AND ‘ductus arteriosus, patent’, combined with ‘indomethacin’ OR ‘ibuprofen’ OR ‘cyclooxygenase inhibitors’ OR ‘paracetamol’. This search revealed a total of 787 hits. We excluded non-randomised studies and RCTs that are not placebo-controlled. Some eligible studies had to be excluded due to language (non-English) or unavailable full text. A total of 32 RCTs were included in a systematic review [15, 18, 44, 52–80]. Data on the outcome parameters were extracted independently by two reviewers (WO and WdB) and entered into Review Manager Software for meta-analysis (Revman version 5.3 Copenhagen: The Nordic Cochrane Centre, The Cochrane Collaboration, 2014). Random effects meta-analysis of the 32 included studies showed that, when compared with placebo, COXi are effective in ductal closure on the short term, since the risk ratio for failure of ductal closure is 0.44 (0.38–0.50). However, this was not associated with a reduction in mortality and morbidity (Table 1).

**Table 1:** Meta-analysis of COXi versus placebo in preterm neonates with PDA

Outcome	Studies	Participants	Risk Ratio	95%-CI
Mortality	31	3534	0.98	0.84–1.13
BPD (total)	23	3531	1.07	0.98–1.16
BPD (oxygen need at PNA 28 days)	16	1395	1.07	0.94–1.22
BPD (oxygen need at PMA 36 weeks)	8	2136	1.06	0.95–1.20
NEC	23	3285	1.05	0.83–1.32
Death or BPD at PMA 36 weeks	7	2096	1.05	0.97–1.14
IVH	20	3150	0.98	0.88–1.10
Failure of ductal closure	23	1619	0.44	0.38–0.50

*CI,* Confidence interval; *BPD,* Bronchopulmonary dysplasia; *PNA,* Postnatal age; *PMA,* Postmenstrual age; *NEC,* Necrotising enterocolitis (any grade); *IVH,* Intraventricular haemorrhage (any grade)

**Fig. 1:**
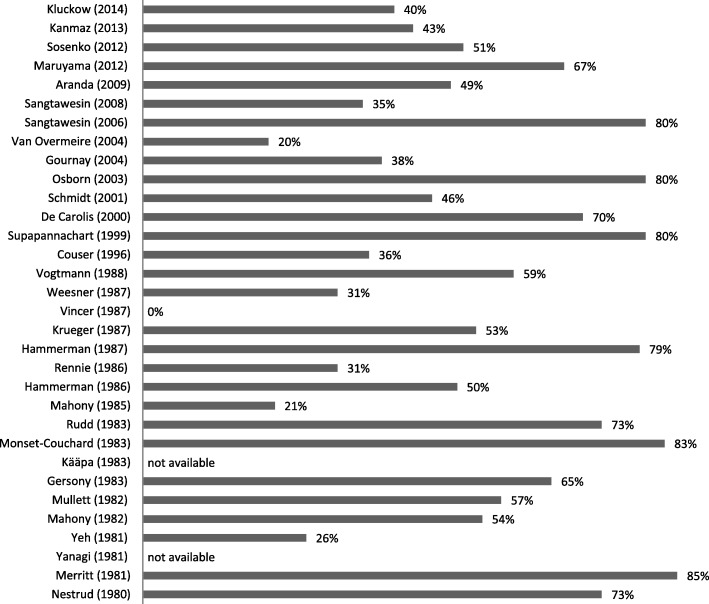
Percentage of patients in the control group eventually treated for their PDA

Based on these data, it has been assumed that PDA treatment, although it does lead to a higher rate of ductal closure, does not lead to a significant better outcome. However, critical analysis of the data shows that a substantial part (up to 85%) of the control group was actually treated for PDA (Fig. 1). So, instead of concluding that PDA treatment does not lead to a better outcome it can only be concluded that there is no significant difference in early versus later or delayed treatment, due to the high amount of treated infants in the control group.

### Randomised controlled trials evaluating expectative management

Until now, no RCT has been published that compares treatment of a PDA with COXi with an expectative approach, i.e. no treatment intended to actively close the PDA. Table 2 gives an overview of recent observational studies describing the outcome of conservative management, that were compared with the Vermont Oxford Network database from 2009 [81–90]. Several studies were excluded due to a high treatment rate in the control group with both INDO (up to 100%) and/or ligation (up to 72%) [39, 91–94]. In addition, the conservative management was rather heterogeneous, ranging from an expectative management to fluid restriction, diuretics and/or adapted ventilator settings. Therefore, although these studies suggest that an expectative approach does not seem to be associated with an increased incidence of neonatal mortality or morbidity, convincing evidence supporting this wait-and-see policy is still lacking, especially in preterm infants born at less than 28 weeks’ gestation.

**Table 2:** Outcome of conservative PDA management in cohort studies compared to the Vermont Oxford Network database 2009 (Horbar et al. (2012))

Studies	Vanhaesebrouck et al. (2007)	Mirea et al. (2012)	Sadeck et al. (2014)	Rolland et al. (2015)	Sung et al. (2016)	Lokku et al. (2017)	Letshwiti et al. (2017)	Slaughter et al. (2017)	Mohamed et al. (2017)	Horbar et al. (2012)
Study design										
Study period	1 Jan 2005–31 Dec 2005	2004–2008	1 Jan 2010–31 Dec 2011	1 Jun 2008–31 Jul 2010	1 Jul 2009–30 Jun 2014	2006–2012	Jan 2004–Feb 2011	1 Jan 2006–31 Dec 2013	1 Jan 2001–31 Dec 2014	2000–2009
Study design	Prospective Monocentre	Retrospective Multicentre	Retrospective Multicentre	Retrospective Monocentre	Retrospective Monocentre	Retrospective Multicentre	Retrospective Monocentre	Retrospective Multicentre	Retrospective Monocentre	Retrospective Multicentre
Compared cohort(s)	CTG vs VON database 2004	CTG vs R_x_ and/or ligation	CTG vs R_x_ and/or ligation	CTG description	CTG vs R_x_ and/or ligation	CTG vs R_x_ and/or ligation	CTG vs STG vs ETG	CTG vs R_x_	CTG vs STG	2009 vs 2000–2008
Total patients	30	3556	494	103	178	5824	371	12,018	643	305,770
Demographics in CTG patients										
Patients	30	577	187	91	97	1486	72	8130	228	43,566 in 2009
Patients with PDA	10	577	187	70	97	1486	34	8130	NA	NA
PDA treatment	0 (0)	0 (0)	0 (0)	1 (1.4)	2 (2.1)	0 (0)	5 (14.7)	0 (0)	NA	NA
Male sex	14 (46.7)	321 (55.6)	91 (48.7)	54 (59.3)	54 (55.7)	811 (54.6)	16 (47.1)	4302 (52.9)	122 (53.5)	51.1% in 2000–2009
Gestational age, in weeks	26.6 [25–30]	28.3 ± 2.3	27.6 ± 2.2	26.3 ± 1.0	24.5 ± 1.0	28.2 ± 2.4	27.4 ± 2.7	≤ 28	28.0 ± 3.4	28.1 in 2009
Birthweight, in grams	994 [600–1484]	NA	772.0 ± 142.3	823 ± 164	718 ± 137	NA	1010 ± 250	NA	1016 ± 340	1055 in 2009
Outcome in CTG patients										
Mortality	(12)	72 (12.5)	96 (51.3)	(17)	9 (9.3)	160 (10.8)	(3)	1067 (13.1)	24 (12.1)	(12.7)
BPD^§^	(7)	138 (27.1)	48 (25.7)	(35)	35 (38)	307 (23.1)	(18)	2509 (30.9)	9 (5.0)	(26.3)^††^
NEC^†^	(0)	34 (6.0)	14 (7.5)^*^	(3)^*^	12 (12.4)	102 (6.9)	(6)^*^	NA	20 (8.8)^*^	(5.3)^††^
IVH^‡^	(2)	105 (21.6)	37 (19.8)	(21)	12 (12.4)	251 (16.9)	(9)	NA	14 (6.6)	(6.1)^††^

Data presented as number n and/or (%), median [interquartile range] or mean ± SD

Percentage may differ due to missing values or lack of assessment

^§^Supplemental oxygen need at a postmenstrual age of 36 weeks, ^†^ Bell stage ≥2, ^‡^ ≥ grade 3, ^*^ no or aberrant definition in article, ^††^ morbidity among survivors (*n* = 38,017)

*CTG* conservative treatment group, *ETG* early treatment group, *STG* symptomatic treatment group, *VON* Vermont Oxford Network; *R*_*x*_ pharmacotherapy, *NEC* Necrotizing enterocolitis, *IVH* Intraventricular haemorrhage, *BPD* Bronchopulmonary dysplasia, *NA* not available

### Research gap

To date, no RCT has been published that compares early treatment of a PDA with COXi in preterm infants less than 28 weeks’ gestation with an expectative approach, that is defined as no intervention in relation to the PDA.

## Methods/design

### Study aims

Our aim is to investigate whether in preterm infants, born at a GA less than 28 weeks, with a PDA (diameter > 1.5 mm) at a PNA < 72 h, an expectative management is non-inferior to early treatment with regard to the composite of mortality and/or NEC (Bell stage ≥ IIa) and/or BPD at a postmenstrual age (PMA) of 36 weeks.

### Study design and settings

**Fig. 2:**
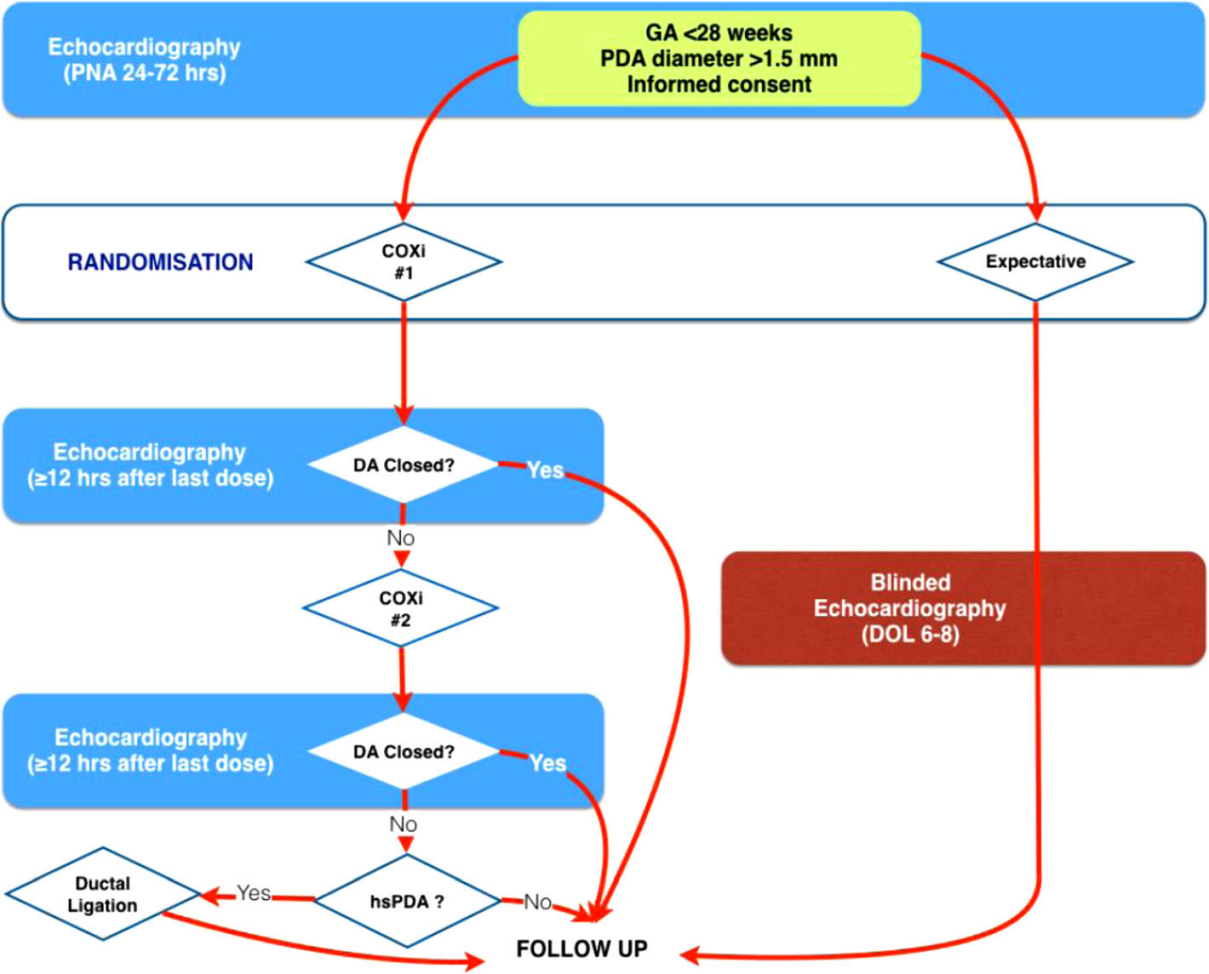
Flow chart of the study design. *COXi,* cyclo-oxygenase inhibitor; *DA,* Ductus arteriosus; *DOL,* day of life; *GA,* gestational age; *(hs)PDA*, (Haemocyamic significant) patent ductus arteriosus; *PNA,* postnatal age

Multicentre, randomised, non-inferiority trial conducted in level III neonatal intensive care units (NICUs) in Europe (BeNeDuctus trial). A flow chart of the study design is shown in Fig. 2.

#### Ethical consideration

After analysis of the results from many RCTs it has been concluded that treatment of a PDA does not result in a decreased rate of mortality and morbidity. A conservative approach towards a PDA is increasingly used in many centres worldwide without a concomitant increase in mortality or morbidity [51, 81–89, 95]. The administration of IBU in the treatment arm of this trial does not pose an extra burden on the patient as it is considered routine treatment in preterm infants with a PDA in many NICUs. Patients who are not treated with IBU are refrained from potential adverse effects of this drug. All patients in this study are treated in accordance with current (inter)national guidelines and local protocols regarding neonatal intensive care management. All primary and secondary outcome parameters are evaluated as part of routine care in Belgium and the Netherlands. No extra investigations, apart from the blinded echocardiogram in the expectative treatment arm, or interventions are needed in this study. Gentle handling of the preterm during echocardiography has been shown not to disturb cardiorespiratory stability [96, 97].

### Definitions

Transductal diameter of a PDA is measured as described by Kluckow and Evans [98]. Of note, the inclusion criterion of a transductal diameter > 1.5 mm is not meant to define hemodynamic significance. It is only used to exclude randomisation of preterm infants with a nearly closed DA. A DA is considered to be closed when the transductal diameter measures less than 0.5 mm or it cannot be visualized using colour Doppler imaging. NEC is classified according to the modified Bell staging criteria [99]. BPD is defined as the need for supplemental oxygen at a PMA of 36 weeks and diagnosed following international standard criteria by Bancalari, including an oxygen reduction test according to Walsh [100, 101]. Hypotension is defined as a mean arterial blood pressure less than the gestational age in weeks. IVH is classified according to the classification by Volpe [102]. Periventricular echogenicity is classified according to the classification by Hashimoto et al. [103]. Sepsis is defined as a positive blood culture for which the patient has been treated with antibiotics. ROP is classified according to the international classification [104].

Preterm infants born at a GA of less than 28 weeks, admitted to a level III NICU, both inborn and outborn, are eligible.

***Inclusion criteria*** are (1) preterm infants born at a GA < 28 weeks; (2) PNA between 24 and 72 h; (3) PDA diameter > 1.5 mm and predominantly left-to-right transductal shunt (≥ 66% of the cardiac cycle); and (4) signed informed consent obtained from parent(s) or representative(s). ***Exclusion criteria*** are (1) contraindication(s) for the administration of IBU (e.g. active bleeding, especially intracranial or gastrointestinal haemorrhage; thrombocytopenia (< 50x10E9/L); renal failure (raised creatinine (> 120 μmol/L) or oliguria (< 0.5 mL/kg/h)); known or suspected NEC); (2) use of COXi prior to randomisation; (3) persistent pulmonary hypertension (ductal right-to-left shunt ≥33% of the cardiac cycle); (4) congenital heart defect, other than PDA and/or patent foramen ovale; (5) life-threatening congenital defects or; (6) chromosomal abnormalities and/or congenital anomalies associated with abnormal neurodevelopmental outcome.

#### Primary outcome definition

The primary endpoint is the composite of mortality, and/or NEC (Bell stage ≥ IIa), and/or BPD at a PMA of 36 weeks.

#### Secondary outcome definition

During the first eleven postnatal days there will be a daily recording in the electronic Case Report Form (eCRF) of the following, first available parameters in the morning: (a) blood pressure (systolic, diastolic and mean pressure) in mmHg; (b) heart rate in beats per minute; (c) urine output in mL/kg/h in the last 8–12 h; (d) actual weight in grams; (e) total daily fluid intake in mL/kg/24 h and; (f) total enteral intake in mL/kg/24 h.

Secondary endpoints are divided in three categories:

1. Short term sequelae of cardiovascular failure, such as (a) hypotension and; (b) need for cardiovascular support.
2. Adverse events during hospitalization, such as (a) BPD at a PNA of 28 days; (b) mortality at a PNA of 28 days and at hospital discharge; (c) modes and duration of respiratory support; (d) total days of oxygen supplementation; (e) incidence of pulmonary air leakage (e.g. pneumothorax); (f) PH; (g) IVH; (h) periventricular echogenicity; (i) NEC; (j) gastrointestinal bleeding; (k) spontaneous intestinal perforation; (l) time to full enteral feeding; (m) sepsis; (n) ROP; (o) adverse effects of IBU; (p) need for surgical ligation of PDA and; (q) length of hospitalization.
3. Neurodevelopmental outcome is assessed in all Dutch and Belgian children in the National Neonatal Follow Up Program at a corrected age of 24 months by (a) paediatric and neurologic examination; (b) cognitive assessment with Bayley Scales of Infant and Toddler Development, Third Dutch Edition (BSID-III-NL); (c) behavioural assessment with Child Behavior Check List (CBCL), Teacher Report Form (TRF) questionnaire and; (d) motor function with Movement Assessment Battery for Children, Second Dutch Edition (Movement ABC 2-NL). For non-Dutch or Belgian children equivalent assessments may be used.

#### Economic evaluation

The economic evaluation is performed along-side the randomised clinical study. We will conduct both a cost-effectiveness analysis (CEA) and a budget impact analysis (BIA).

### Cost-effectiveness analysis

The potential efficiency of expectative management of PDA in preterm infants with a PDA is compared to the heterogeneous usual care for preterm infants with a PDA. The CEA is performed from a societal perspective. We hypothesize that expectative management is the cost-effective alternative, because it saves on medical treatments and diagnostics at non-inferior effectiveness. The economic evaluation is based on the general principles of a CEA. Primary outcome measures for the economic evaluation, considering the 24 months follow-up period, are (in)direct costs and composite of survival and/or NEC and/or BPD. When this composite does not differ between an expectative management and usual care the cost-effectiveness decision rule will be cost minimization, else it will be cost associated with a gain or loss in survival and/or NEC and/or BPD. This efficiency outcome will be computed and uncertainty will be determined using the bootstrap method. If a difference between the two alternative treatments occurs, a cost-effectiveness acceptability curve will be derived that is able to evaluate efficiency by using different thresholds (Willingness To Pay) for a combined survival effect. The impact of uncertainty surrounding deterministic parameters on the efficiency outcome will be explored using one-way sensitivity analyses on the range of extremes.

The cost analysis exists of two main parts. First, on patient level, volumes of care will be measured prospectively over the time path of the clinical study using the eCRF and/or medical records and the inpatient treatment facilities administration system to collect information on for example: consultation paediatric cardiologist, echocardiography, chest X-ray, medication, intensive care transport and ductal ligation. Second per arm full cost-prices will be determined using the Dutch guideline [105], or else real cost prices via activity based costing or centre-specific cost information. Productivity losses for parents will be estimated using a patient-based iMTA Productivity Cost Questionnaire adapted to parents at a postnatal age of 4 weeks and a corrected age of 6, 12 and 24 months [106]. The questionnaire is given to the parents by mail together with a post-paid envelope or sent via electronic mail. The friction cost-method will be applied following the Dutch guidelines [105]. The cost analysis will be performed using a mixed model approach with centre as random coefficient and potential confounders as fixed.

### Budget impact analysis

The aim of this BIA is to assess the financial consequences of implementing an expectative management in the Dutch health care system in the short-to-medium term from the budget holder’s perspective [107]. The BIA base-case perspectives are respectively societal, health insurance/third party payer and health care. A global average cost per patient for expectative management is €89,000 and for the usual care €92,000. Multiplied by the yearly number of preterm neonates with a PDA in the Netherlands (*n* = 270) gives a global impression of the magnitude of the budget impact, namely €24,000,000 compared to €24,800,000. This provides a yearly budgetary saving of about €800,000. At least four scenarios will be considered, namely (1) current care; (2) immediate 100% expectative management; (3) gradual implementation of expectative management and; (4) partial implementation of expectative management. The BIA will be assessed through (decision analytical) modelling and analysed, if possible, in a probabilistic way [108].

#### Randomisation process

In the absence of exclusion criteria, eligible patients will be randomised to either the expectative management arm or the medical treatment arm. The randomisation is coordinated centrally and web-based. Randomisation will be per centre and stratified according to GA stratum (Stratum A: GA < 26^0/7^ weeks; Stratum B: GA 26^0/7^–27^6/7^ weeks). The block size will vary in a range from four to eight. The intention is to randomise multiple birth infants independently, unless there is an explicit request from the parents/caretakers to expose the siblings to the same treatment.

#### Withdrawal and replacement of individual subjects

The investigator or attending physician can decide to withdraw a subject from the study for urgent medical reasons. If they wish, parents or caregivers can leave the study at any time for any reason. Only patients that are withdrawn from the study at the request of parents or caregivers will be replaced. The total number of patients that can be replaced is limited to twenty-five. Infants who are withdrawn from the study, will receive standard of care, including regular follow up after discharge, with assessment of neurodevelopmental outcome. Patients in the expectative management arm that meet the criteria for open label treatment with IBU (Table 3) and/or surgical ligation (Table 4) will remain in follow up and are therefore not withdrawn from the study.

**Table 3:** *Open label* criteria

I. Exclusion of other causes of cardiovascular failure (e.g. sepsis or congenital heart defect)	
AND	
II. Clinical findings of cardiovascular failure secondary to significant ductal left-to-right shunting: a. Signs of systemic hypoperfusion (refractory systemic hypotension and/or elevated serum lactate concentration (> 2.5 mmol/L)) and; b. Signs of pulmonary hyperperfusion (prolonged ventilator dependency).	
AND	
III. Echocardiographic findings of significant ductal left-to-right shunting a. Diameter of PDA > 1.5 mm, and; b. Unrestricted ductal left-to-right shunting (‘pulsatile pattern’): end-diastolic flow velocity < 50% of peak flow velocity, and; c. End-diastolic flow velocity left pulmonary artery > 0.3 m/s, and; d. Left atrial to aortic ratio > 1.5. AND a. Severe left ventricular failure (mitral regurgitation), and; b. Disturbed end-organ perfusion (retrograde diastolic blood flow in descending aorta).

**Table 4:** Ligation criteria

I. Exclusion of other causes of cardiovascular failure (e.g. sepsis or congenital heart defect)	
AND	
II. Clinical findings of cardiovascular failure secondary to significant ductal left-to-right shunting: a. Signs of systemic hypoperfusion (refractory systemic hypotension and/or elevated serum lactate concentration (> 2.5 mmol/L)) and/or; b. Signs of pulmonary hyperperfusion (prolonged ventilator dependency).	
AND	
III. Echocardiographic findings of significant ductal left-to-right shunting a. Diameter of PDA > 1.5 mm, and; b. Unrestricted ductal left-to-right shunting (‘pulsatile pattern’): end-diastolic flow velocity < 50% of peak flow velocity, and/or; c. End-diastolic flow velocity left pulmonary artery > 0.3 m/s, and/or; d. Left atrial to aortic ratio > 1.5. AND/OR a. Severe left ventricular failure (mitral regurgitation), and/or; b. Disturbed end-organ perfusion (retrograde diastolic blood flow in descending aorta).	

### Treatment arms

#### Expectative management arm (intervention)

Patients randomised to the expectative management arm will not receive COXi, including for indications other than closure of the DA. No (additional) putative interventions to prevent or treat a PDA, for example fluid restriction or diuretics for that purpose only, are allowed. When the attending physician thinks that the patient is in danger when being deprived from treatment with COXi, *open label* treatment can only be considered when pre-specified criteria are met (Table 3). To be informed about the natural course of ductal closure echocardiography is performed at the end of the first week of life, but only when it is feasible for the clinical team to remain blinded for the results.

#### Medical treatment arm (control)

Patients in the medical treatment arm receive COXi as soon as possible after randomisation, preferably within 3h. In this study IBU is used, because it seems to be as effective in ductal closure in preterm infants as INDO. Besides, IBU might have less side-effects than INDO, since IBU reduces the risk of NEC and transient renal insufficiency [17], does not affect mesenteric blood flow, has less effect on renal perfusion [109–111], and influences cerebral blood flow in a lesser extent [111–114]. The dosing scheme for IBU is according to local guidelines. The preferred route of administration of IBU is intravenously. However, this is at the discretion of the attending physician, since enteral administration appears at least as effective [17, 115–118].

Echocardiographic re-evaluation is performed at least 12h after the last (third) dose of the first IBU course. If the DA is found to be closed, no further analysis or treatment is needed regarding the DA. When the DA has not closed, a second course of IBU is started at least 24h after the third dose of the first course, in a similar dosage. 12 to 24h after the last (sixth) dose of the second course echocardiography is performed again. If the DA is found to be closed, no further analysis or treatment is needed regarding the DA. When the DA failed to close after two courses of IBU and is still classified as a hsPDA, ductal ligation can be considered, when the ligation criteria are met (Table 4).

#### Co-interventions

It is essential that neonatal management is similar in both study arms except for the prescription of IBU and routine echocardiography at the end of the drug course(s) in the medical treatment arm. All patients in this study will be treated according to current (inter)national guidelines and local protocols regarding neonatal intensive care management. When ductal closure has not been documented before discharge, ductal patency is echocardiographically examined in both arms of the study, when this is indicated by the local paediatric cardiologist and only at a date after the primary outcomes have been established, after a postmenstrual age of 36 weeks. Echocardiographic pictures and movies are stored and collected for blinded re-analysis at the end of the study.

All prognostic relevant co-interventions and conditions will be documented, using the standard medical records, such as (a) administration of antenatal steroids; (b) maternal disease (e.g. pre-eclampsia); (c) maternal medication, especially COXi; (d) mode of delivery; (e) multiple birth; (f) duration of rupture of membranes; (g) GA at birth; (h) birth weight; (i) Apgar scores at 5min; (j) umbilical blood gas analysis; (k) resuscitation after birth; (l) surfactant administration, and; (m) postnatal steroids.

## Sample size, power and statistical methods

### Sample size

Based on data from the Dutch Perinatal Registry the incidence of our primary outcome measures mortality, NEC and BPD is 20, 10 and 15% respectively in preterm infants less than 28 weeks’ gestation [119]. Non-inferiority is defined as a significant difference in the primary outcome parameter between the two arms of less than 10%. In other words, the 95% confidence interval of the observed difference between an expectative approach and COXi treatment should not exceed the non-inferiority margin of 10%. With an estimated a priori risk for the composite of mortality and/or NEC and/or BPD at 36 weeks PMA of 35%, a one sided type I error of 5% and a power of 80%, the sample size to exclude a non-inferiority margin of 10% for the difference of proportion of participants reaching the primary outcome parameter is 564 patients, being 282 patients in each arm. This sample size was calculated using PASS 2008, version 08.0.8 NCSS.

### Time frame

Based on retrospective data a total of 540 preterm neonates with a GA less than 28 weeks will be born yearly in The Netherlands, of whom approximately 270 (50%) will have a PDA at a PNA of 24–72 h. With an estimated inclusion rate of 66% (*n* = 178), patient recruitment will take approximately 3 years.

#### Data analysis

Treatment effects for the dichotomous clinical outcomes will be reported using risk differences with 95% confidence interval. Normally distributed data will be presented as mean ± standard deviations, uneven distributed data as medians with interquartile ranges. Categorical data will be analysed using the Chi-square for two- and multiway tables. Continuous data will be analysed using the Student’s t test. Both intention-to-treat and per-protocol analyses will be employed. Statistical significance is defined as a *p*-value < 0.05. For the primary outcome a 95% one sided confidence interval for the risk difference will be calculated and when based on this interval a difference of 10% or more can be excluded, non-inferiority will be concluded.

## Adverse events and monitoring

### Data safety monitoring board

An external Data Safety Monitoring Board (DSMB) will monitor the safety, validity, and credibility of the trial in order to protect the patients and will provide the trial’s Steering Committee with recommendations regarding continuation or cessation of the trial. The normal distribution between the components of the primary outcome parameter will be closely monitored by the DSMB. The DSMB is composed of three individuals: a neonatologist with extensive knowledge about PDA, a statistician who has experience with clinical trials and a paediatric cardiologist with extensive knowledge about neonatal haemodynamics. The composition, tasks, responsibilities and working procedures of the DSMB are described in a charter. The DSMB will meet to discuss the findings of the safety interim analyses. These will be conducted when 15, 30, 50 and 75% of the data have been gathered.

The DSMB charter states that there are two possible reasons for stopping the study early, namely concerns for safety and futility. In principle, the trial will not be stopped early before the minimum number of evaluable patients required (*n* = 564) are included for beneficial effect of IBU treatment on the primary outcome. Unless there is an unacceptably high rate of mortality in either the IBU or expectative group, this is to preserve the power for evaluation of neurodevelopmental outcome at 2 years corrected age. Hence, the interim analyses will not be associated with alpha spending.

### Reporting adverse events

Adverse events are defined as any undesirable experience occurring to a subject during the study, whether or not considered related to the interventions in this study. All adverse events observed by the parents, caretakers or the investigator and staff will be recorded in the eCRF until discharge home.

A serious adverse event (SAE) is any untoward medical occurrence or effect that at any dose (a) results in death; (b) is life threatening (at the time of the event); (c) requires hospitalization or prolongation of existing inpatients’ hospitalization; (d) results in persistent or significant disability or incapacity, and; (e) is a congenital anomaly or birth defect (not applicable in this study). Any other important medical event that may not result in death, be life threatening, or require hospitalization, may be considered a SAE when, based upon appropriate medical judgement, the event may jeopardize the subject or may require an intervention to prevent one of the outcomes listed above. An elective hospital admission will not be considered a SAE.

All SAEs will be reported, by the coordinating principle investigator (PI) to the DSMB and through the web portal ToetsingOnline to the accredited medical ethics committee (MEC) that approved the protocol. In non-Dutch centres the PI will report to the coordinating PI in The Netherlands and to the relevant national authorities. All adverse events will be followed until they have abated, or until a stable situation has been reached. SAEs need to be reported till end of study.

This study population has a high risk of serious complications, which are inherent to their vulnerable condition and unrelated to the intervention which is under evaluation in this trial, the so-called ‘context-specific SAEs’. These are included in the primary and secondary outcomes of this study and are recorded in the eCRF by the PI. Immediate and individual reporting of all these condition related complications will not enhance the safety of the study, so they will be presented to the DSMB and MEC once a year [120–122].

### Current status of trial

The first patient has been included in the study in December 2016.

## Discussion

A growing number of clinicians believe the PDA is an innocent bystander, since no causal relationship has been proven between a hsPDA and the risk of conditions related to pulmonary hyperperfusion (e.g. PH and BPD) and/or systemic hypoperfusion (e.g. NEC). An expectative management is gaining interest, although convincing evidence to support this management is lacking, since there is no RCT available comparing treatment with an expectative approach. We found only one small study describing a prospective cohort and several retrospective studies comparing two or three time eras with comparison of different management approaches in preterm infants with a persistent PDA [81–89]. These observational studies have not shown a concomitant increase in mortality and morbidity related to a decrease in active ductal closure.

In this study we randomise preterm infants born at less than 28 weeks’ gestation to two different intentions regarding the management of a PDA. Our primary hypothesis is that an expectative treatment is non-inferior to early treatment of a PDA in premature infants born at a GA less than 28 weeks. In the treatment arm the PDA is regarded a plausible cause of neonatal mortality and morbidity secondary to an increased pulmonary perfusion at the expense of systemic hypoperfusion, while in the expectative management arm the PDA is accepted as a non-pathological phenomenon and PDA is merely regarded as a marker of immaturity. It was deliberately chosen not to perform a placebo-controlled trial, because it is our conviction that then the focus would be on treatment of a PDA in the study population with an associated increased risk of open label treatment, as has occurred in former RCTs. To further minimize the risk of contamination of the expectative management group we defined strict open label criteria.

We aim to gain more insight in the natural course of the PDA in the expectative management arm. Therefore, an echocardiogram, that is blinded for the attending clinical team, is performed at the end of the first week. This trial will be protected from selection bias by using concealed, stratified and blocked randomisation. Patient characteristics will be collected from all eligible infants that are not included in this study in order to assess any potential recruitment bias.

If this trial supports our hypothesis that an expectative management is non-inferior to early closure, there will be a reduction in costs, which will be calculated with the CEA en BIA. Not only in this economic perspective an expectative treatment would be more interesting, also vulnerable premature infants will be prevented from potential adverse effects from medical or surgical treatment.

## Abbreviations

BIA: Budget impact analysis
BPD: Bronchopulmonary dysplasia
CEA: Cost effectiveness analysis
COXi: Cyclooxygenase inhibitors
DA: Ductus arteriosus
DSMB: Data safety monitoring board
eCRF: Electronic case report form
GA: Gestational age
hsPDA: Haemodynamically significant patent ductus arteriosus
IBU: Ibuprofen
INDO: Indomethacin
IVH: Intraventricular haemorrhage
MEC: Medical ethics committee
NEC: Necrotising enterocolitis
NICU(s): Neonatal intensive care unit(s)
PDA: Patent ductus arteriosus
PH: Pulmonary haemorrhage
PI(s): Principle investigator(s)
PMA: Postmenstrual age
PNA: Postnatal age
RCT(s): Randomised controlled trial(s)
ROP: Retinopathy of prematurity
SAE(s): Serious adverse event(s)
